# Supplementary material for: Seminoma and Embryonal Carcinoma Footprints Identified by Analysis of Integrated Genome-Wide Epigenetic and Expression Profiles of Germ Cell Cancer Cell Lines
Source: PLoS One. 2014 Jun 2;9(6):e98330. doi: 10.1371/journal.pone.0098330 (PMC4041891; doi:10.1371/journal.pone.0098330)
Supplement: File S1 — ZIP file containing DMRforPairs output for significant regions. Please start from the html files. (ZIP) [file pone.0098330.s008.zip › figures/19150.pdf]

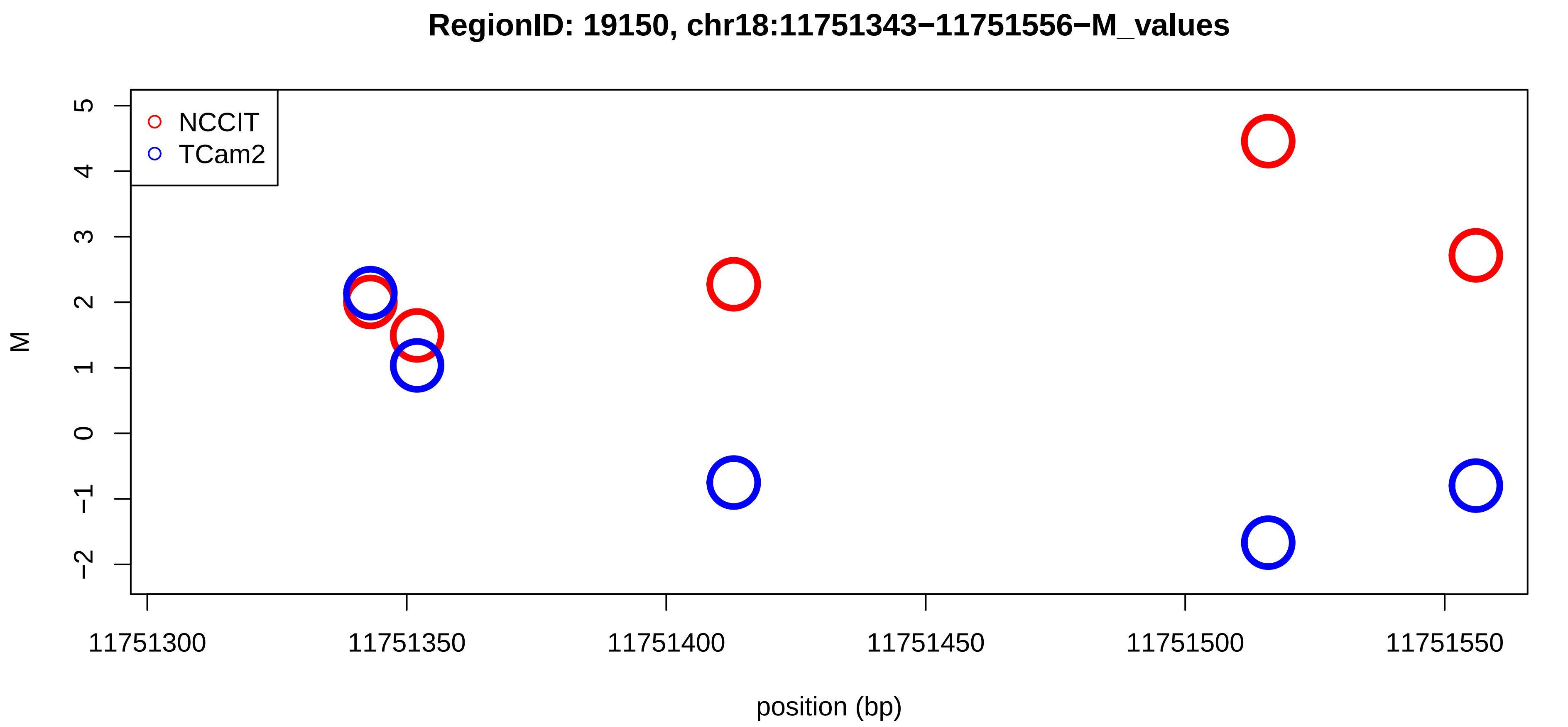
RegionID: 19150, chr18:11751343−11751556−M_values
NCCIT
TCam2
M
5
4
3
2
1
0
−1
−2
11751300
11751350
11751400
11751450
11751500
11751550
position (bp)

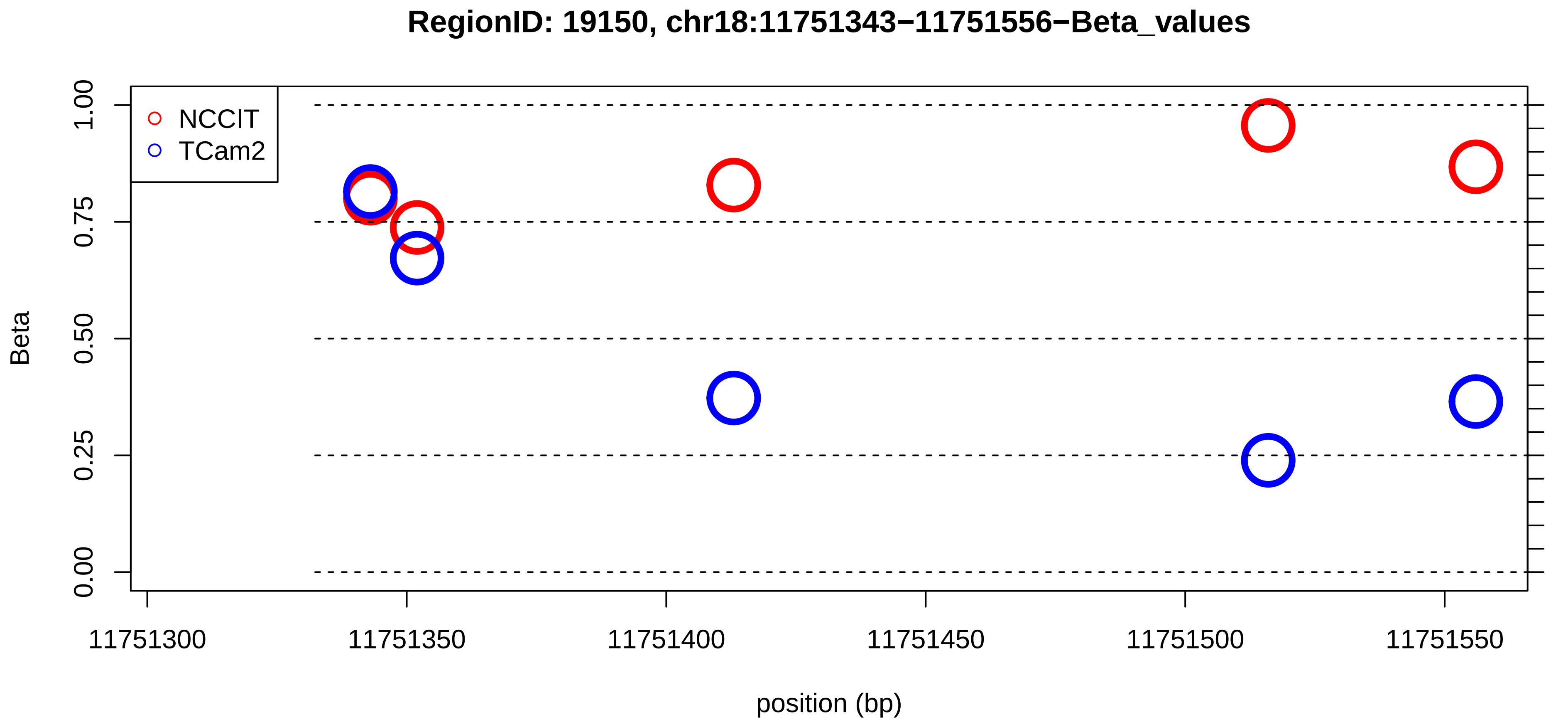
RegionID: 19150, chr18:11751343−11751556−Beta_values
NCCIT
TCam2
Beta
1.00
0.75
0.50
0.25
0.00
11751300
11751350
11751400
11751450
11751500
11751550
position (bp)
